# Effectiveness and safety analysis of initial treatment with belimumab in childhood-onset systemic lupus erythematosus

**DOI:** 10.3389/fimmu.2026.1769627

**Published:** 2026-03-16

**Authors:** Yan Li, Jing Ma, Mengyue Deng, Qin Ma, Ziwei He, Tongxin Han, Yurong Piao, Fei Sun, Zhou Shu, Wenxiu Mo, Jiapeng Sun, Siqi Du, Ling Bai, Ziweidiguli Maimaiti, Huawei Mao

**Affiliations:** 1 Department of Immunology, Ministry of Education Key Laboratory of Major Diseases in Children Beijing Key Laboratory of Precision Diagnosis and Treatment of Pediatric Immune Diseases, Beijing Children’s Hospital, Capital Medical University, National Center for Children’s Health, Beijing, China; 2 Department of Nephrology & Rheumatology, Xinjiang Hospital of Beijing Children’s Hospital, Children’s Hospital of Xinjiang Uygur Autonomous Region, Urumqi, Xinjiang, China; 3 Capital Medical University, Beijing, China

**Keywords:** belimumab, child, glucocorticoid, infection, initial treatment, systemic lupus erythematosus

## Abstract

**Objective:**

A retrospective cohort study to analyze the effectiveness and safety of belimumab using in the initial treatment of childhood-onset systemic lupus erythematosus (cSLE).

**Methods:**

We collected clinical data from all children with a first diagnosis of cSLE admitted to our center between 1 April 2021 and 1 November 2024. Patients who initiated belimumab within 1 month of diagnosis were assigned to the belimumab group, and those who did not receive belimumab comprised the control group. Propensity score matching (PSM) was applied to balance baseline characteristics between the groups. The proportion of lupus low disease activity status (LLDAS) and remission (Definitions of Remission in Systemic Lupus Erythematosus, DORIS), the disease activity scores, laboratory tests, glucocorticoid dosage, and adverse effects during the courses of treatment in two groups were analysis.

**Results:**

There were 39 cases in both the belimumab group and the control group. The belimumab group exhibited a higher proportion of patients achieving LLDAS (31/39 vs. 14/39, p < 0.001) and DORIS (18/39 vs. 5/39, p =0.002) compared to the control group at 12 months after treatment. Additionally, the time to achieve LLDAS and DORIS was significantly shorter in the belimumab group (log-rank p< 0.001). However, no statistical variances were observed between the two groups in Systemic Lupus Erythematosus Disease Activity Index (SLEDAI) scores, physician global assessment (PGA) scores, complement levels, negative rates of anti-double-stranded DNA (anti-dsDNA) antibodies at each follow-up interval. From the 7th to the 12th month of treatment, the daily prednisone dose in the belimumab group was lower than in the control group. After 12 months of treatment with belimumab, B cells (p< 0.001) and immunoglobulin G (IgG) (p< 0.001) showed a significant decrease from baseline. No infusion-related adverse reactions were observed in children receiving belimumab, and the infection rate did not differ significantly from the control group.

**Conclusions:**

Adding belimumab to initial therapy facilitates quicker disease control and expedites glucocorticoid tapering in children, which can be a new treatment strategy for cSLE.

## Introduction

Systemic lupus erythematosus (SLE) is an autoimmune disorder marked by multisystem involvement and serum autoantibodies, ranking among the most prevalent pediatric rheumatologic diseases. Childhood-onset SLE represents 20% of cases, with a prevalence of 0.28-0.48 per 100,000, and some cases are linked to gene mutations (1–3). Compared to adult SLE patients, cSLE presents with a more rapid onset, accelerated progression, more severe organ involvement, and a poorer prognosis (4–6). The 2019 European League Against Rheumatism (EULAR) guidelines specify that the primary treatment objective for SLE is to achieve remission or LLDAS, prevent relapse across all organ systems, and minimize prednisone dosage (7). The standard treatment for cSLE currently involves a combination of glucocorticoid, immunosuppressants, and antimalarials (8). Prolonged glucocorticoid use can result in many adverse effects, including infection, osteoporosis, hypertension, hyperglycemia, peptic ulcers, cataracts, and glaucoma (9).

Belimumab, a fully humanized IgG1λ monoclonal antibody targeting B-cell activating factor (BAFF), inhibits B-lymphocyte proliferation and differentiation, thereby reducing autoantibody production (10). In April 2019, the Food and Drug Administration (FDA) approved belimumab for treating cSLE, marking it as the sole biologic approved for aiding disease management and prednisone tapering in cSLE patients (11, 12). Belimumab has traditionally been reserved for cases of poor response to conventional therapy or recurrent SLE (13), with limited research on its use as an initial treatment for SLE. Recent adult studies indicate that early initiation of belimumab improves outcomes, with higher rates of achieving LLDAS and DORIS and faster glucocorticoid tapering (14, 15). Children with cSLE experience a more aggressive disease course and greater cumulative glucocorticoid exposure, so early belimumab intervention may offer distinct advantages in this population. However, data on belimumab as first-line therapy in pediatric patients remain limited. This retrospective cohort study evaluates the effectiveness and safety of adding belimumab to conventional therapy as initial treatment for cSLE, with primary focus on treat-to-target outcomes (LLDAS and DORIS), glucocorticoid-sparing effects, and immunological changes.

## Materials and methods

### Study design and patients

This was a single-center, non-randomized, retrospective cohort study with PSM to compare outcomes in children with newly diagnosed cSLE who received belimumab as part of initial therapy to those who received conventional therapy alone. The study evaluated treatment regimens for cSLE. Conventional therapy included glucocorticoids at an initial dose of 2 mg/kg (converted to prednisone equivalent dose), immunosuppressants (mycophenolate mofetil [MMF], cyclophosphamide [CTX], methotrexate, cyclosporine), and hydroxychloroquine (HCQ). In the belimumab group, belimumab was added to standard of care (SoC) within one month of SLE diagnosis, administered intravenously at 10 mg/kg. The first three doses were given two weeks apart, followed by doses every four weeks.

Clinical data of all pediatric patients diagnosed with SLE at our institution from 1 April 2021 to 1 November 2024 were gathered. Inclusion criteria of patients in our study comprised: 1) age ≤18 years; 2) fulfillment of the classification criteria for SLE established by the EULAR/American College of Rheumatology (ACR) in 2019 (16); 3) initial diagnosis; 4) minimum 12-month follow-up period; 5) availability of complete follow-up information. Exclusion criteria encompassed: 1) overlap syndrome; 2) severe uncontrolled infection; 3) prior belimumab usage. Patient eligibility for belimumab was evaluated within one month of diagnosis. The decision to initiate belimumab was based on clinical judgment, disease activity, organ involvement, and the anticipated need for rapid tapering of steroids. The rationale encompassed moderate to high disease activity (SLEDAI ≥7), significant organ involvement (such as lupus nephritis [LN] or neuropsychiatric lupus), and concerns regarding glucocorticoid-related adverse effects. Patients were not randomly assigned. Instead, they were naturally categorized into two cohorts based on the treatment received: those who were administered belimumab within one month of diagnosis (belimumab group) and those who did not receive belimumab (control group). To mitigate selection bias and potential confounding by indication, PSM was employed to balance baseline characteristics, including gender, age of onset, SLEDAI scores, PGA scores, and initial prednisone dose, between the groups. Our study was approved by the ethics committee of Beijing Children’s Hospital (File number: [2025]-E-062-R), and the legal guardians of the children all gave informed consent and signed the informed consent form.

### Outcome

The study’s primary outcomes included the percentage of children achieving LLDAS and DORIS after 6 and 12 months of treatment, as well as the time taken to reach LLDAS and DORIS in both cohorts. Secondary outcomes encompassed SLEDAI and PGA scores, complement C3 and C4, anti-dsDNA antibody negativity, and daily prednisone dosage to evaluate the effectiveness of the initial treatment combined with belimumab at various time points in both groups. Additionally, changes in weight, height, and body mass index (BMI) from baseline to 12 months were analyzed to assess the impact of glucocorticoids on children’s growth and development. Within the belimumab group, the influence of belimumab on immune function was evaluated by examining lymphocytes, T cells, CD4+ T cells, CD8+ T cells, B cells, naive B cells, memory B cells, transitional B cells, plasmablasts, natural killer (NK) cells and IgG levels at baseline and after 12 months of treatment. A retrospective case review was conducted to summarize adverse effects observed during belimumab treatment and to evaluate its safety as an initial therapeutic approach.

SLEDAI assesses disease activity over the past 10 days. Scores ≤6 indicate mild activity, 7–12 moderate activity, and >12 severe activity (17). PGA is a visual analogue scale reflecting disease activity in SLE. A score of 0 signifies no activity, 0.5 to 1 mild activity, 1 to 2 moderate activity, and 2 to 3 severe activity (18). LLDAS is defined as SLEDAI score ≤ 4, no disease activity in major organs, no new disease activity, PGA score ≤1, prednisone dose ≤7.5mg/day, and standard maintenance doses of immunosuppressants and biologics (19). DORIS defines remission as a clinical SLEDAI of 0 (irrespective of serological parameters), PGA score <0.5, prednisone dose ≤ 5 mg/day, and stable immunosuppressants and biologics (20).

### Statistical analyses

SPSS26.0 was used for data statistical analysis. The data that conformed to the normal distribution were expressed as mean ± standard deviation, and comparisons between different groups were performed using the t-test. The data that did not conform to the normal distribution were expressed as median (P25, P75), and comparisons between different groups were performed using the nonparametric test. The normality was tested by the Shapiro‐Wilk test. The count data were expressed as percentages [n (%)], and comparisons between different groups were performed using the Pearson’s χ2 test or Fisher’s exact test. P<0.05 indicated that the difference between the two groups was statistically significant. To ensure baseline comparability between groups, PSM was performed using the MatchIt package (21) in R (version 4.3.1). The propensity score was calculated using logistic regression analysis, incorporating age at diagnosis, gender, PGA, SLEDAI score, and initial steroid dose as covariates. Patients in the belimumab group were matched in a 1:1 ratio to those in the control group using nearest neighbor matching with a caliper width of 0.2 standard deviations of the logit of the propensity score. The balance of baseline covariates before and after PSM was assessed using standardized mean differences (SMDs). An SMD <0.1 was considered indicative of adequate balance. Because the primary objective was to compare outcomes between the two treatment groups at specific clinical timepoints (e.g., 6 and 12 months), we performed pointwise comparisons at each follow-up visit. A multivariable logistic regression analysis was subsequently performed on the matched cohort to adjust for potential residual confounding, incorporating renal involvement, central nervous system (CNS) involvement, and the use of HCQ, CTX, and MMF. Odds ratios (OR) with 95% confidence intervals (CI) were calculated.

## Results

### Baseline characteristics

**Figure 1:**
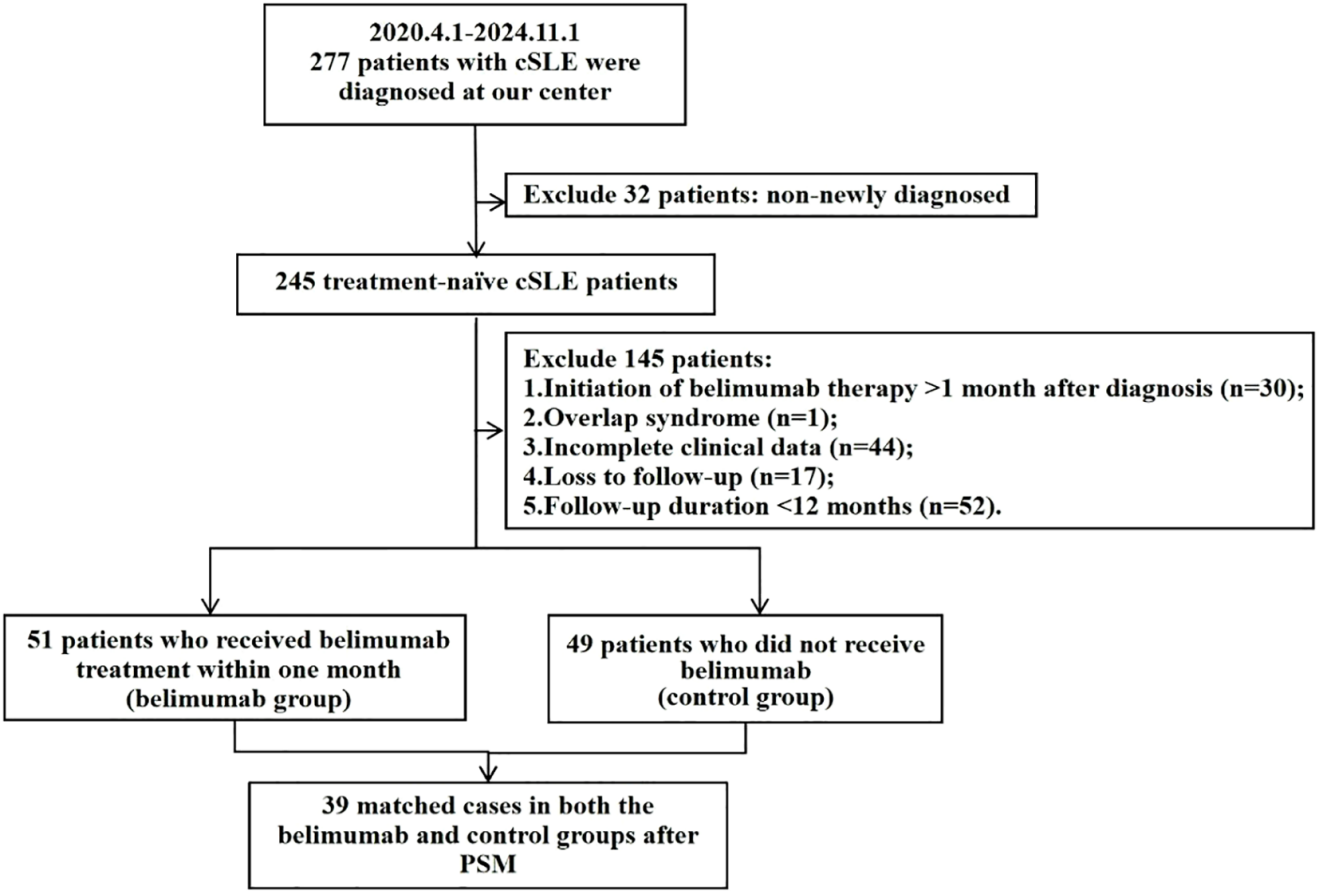
Flow diagram of patient selection and group allocation.

From 1 April 2021 to 1 November 2024, a total of 277 patients with cSLE were enrolled at our center, including 245 newly diagnosed cases. Based on predefined inclusion and exclusion criteria, 51 children who received belimumab treatment within one month were ultimately selected, while 49 children did not receive belimumab (**Figure 1**). PSM was conducted, resulting in 39 matched cases in both the belimumab and control groups based on gender, age at onset, PGA score, SLEDAI score, and initial prednisone dosage. Following PSM, most baseline characteristics were well-balanced (SMD<0.1), though some residual imbalance remained. (**Table 1**). The median number of belimumab administrations in the belimumab group was 17 (14, 21). The median follow-up duration was 15 (13, 19) months in the belimumab group and 23 (15, 40) months in the control group.

**Table 1:** Baseline demographic, clinical, and laboratory characteristics of children with newly diagnosed cSLE in the belimumab and control groups before and after PSM.

Variable	Before PSM			Before PSM		
	Belimumab group	Control group	SMD	Belimumab group	Control group	SMD
N	51	49		39	39	
Gender (female/N)	47/51	39/49	0.299	34/39	33/39	0.074
Age at diagnosis (years)	11.32 ± 2.46	11.83 (9.84, 13.42)	0.015	11.27 ± 2.40	11.30 ± 2.92	0.013
SLEDAI score	12.65 ± 6.14	11.49 ± 4.76	0.211	11.77 ± 6.19	11.46 ± 4.82	0.055
PGA score	2.5 (2.2, 2.5)	2.5 (2.2, 2.6)	0.165	2.50 (2.20, 2.50)	2.50 (2.20, 2.60)	0
Involved systems						
Hematologic involvement	37/51	37/49	0.068	30/39	32/39	0.122
Rash	33/51	35/49	0.145	25/39	29/39	0.224
Kidney	25/51	16/49	0.338	19/39	11/39	0.431
Arthritis	24/51	19/49	0.168	16/39	17/39	0.052
Central nervous system	9/51	6/49	0.152	9/39	5/39	0.27
Laboratory tests						
Anti-dsDNA antibody negativity rate	12/51	14/49	0.115	7/39	12/39	0.302
Complement C3	0.41 (0.22, 0.65)	0.39 (0.28, 0.64)	0.247	0.37 (0.23, 0.63)	0.39 (0.28, 0.64)	0.272
Complement C4	0.04 (0.03, 0.09)	0.12 (0.09, 0.16)	0.124	0.043 (0.03, 0.085)	0.054 (0.039, 0.14)	0
Medication						
Initial prednisone dosage	60 (50, 60)	60 (50, 60)	0.075	60.00 (50.00, 60.00)	60.00 (50.00, 60.00)	0.068
Mycophenolate mofetil (only)	30/51	33/49	0.177	23/39	25/39	0.106
Cyclophosphamide	21/51	14/49	0.267	16/39	12/39	0.215
Hydroxychloroquine	47/51	38/49	0.349	36/39	29/39	0.409

CTX, mycophenolate mofetil; HCQ, hydroxychloroquine; MMF, mycophenolate mofetil; PGA, physician global assessment; PSM, propensity score matching; SLEDAI, Systemic Lupus Erythematosus Disease Activity Index; SMD, standardized mean differences.

### Outcome

#### Primary outcome

**Figure 2:**
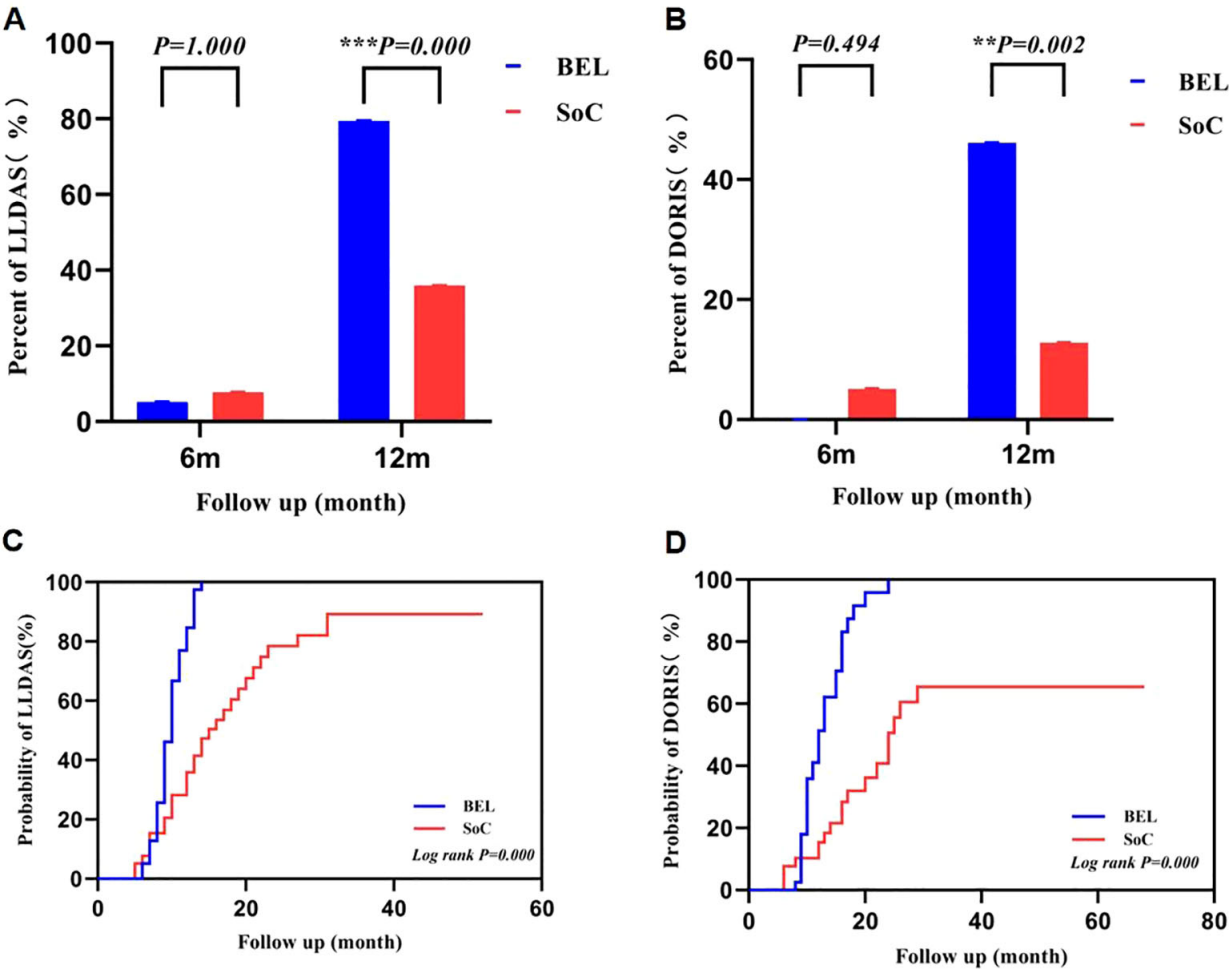
Differences in LLDAS and DORIS between belimumab and control groups. **p < 0.01;***P<0.001; **(A, B)** There were no significant differences in the proportions of LLDAS and DORIS between the belimumab and control groups at 6 months. However, after 12 months of treatment, the belimumab group exhibited significantly higher proportions of LLDAS and DORIS compared to the control group, with a statistically significant difference. **(C, D)** the time to achieve LLDAS and DORIS was significantly shorter in the belimumab groups compared to the control group. LLDAS, lupus low disease activity status; DORIS, Definitions of Remission in Systemic Lupus Erythematosus.

There were no statistically significant differences in achieving LLDAS (Bel 2/39 vs. SoC 3/39, p =1.000) and DORIS (Bel 0/39 vs. SoC 2/39, p =0.494) between the groups at 6 months post-treatment. However, at 12 months post-treatment, the belimumab group demonstrated significantly higher proportions of LLDAS (Bel 31/39 vs. SoC 14/39, p < 0.001) and DORIS (Bel 18/39 vs. SoC 5/39, p=0.002) compared to the conventional treatment group, with statistically significant differences (**Figures 2A, B**). Analysis of the component criteria (**Supplementary Table 1**) showed that the higher rates of LLDAS and DORIS in the belimumab group were mainly attributable to a greater proportion of patients reaching the glucocorticoid dose thresholds (prednisone ≤7.5 mg/day and ≤5mg/day), while achievement of the disease activity components was similar between groups. Furthermore, the belimumab group exhibited a higher rate of attaining LLDAS and DORIS throughout the follow-up period compared to the control group (LLDAS: 39/39 vs.30/39, p=0.002; DORIS: 33/39 vs. 19/39, p=0.001). Additionally, the time taken to reach LLDAS and DORIS was significantly shorter in the belimumab group than in the control group, representing a statistically significant distinction (**Figures 2C, D**). After multivariable adjustment for key clinical covariates, early belimumab treatment remained significantly associated with higher odds of achieving LLDAS (adjusted OR 6.52, 95% CI 2.15–19.81, p=0.001) and DORIS (adjusted OR 6.13, 95% CI 1.74–21.58, p=0.005) at 12 months.

#### Secondary outcome

**Figure 3:**
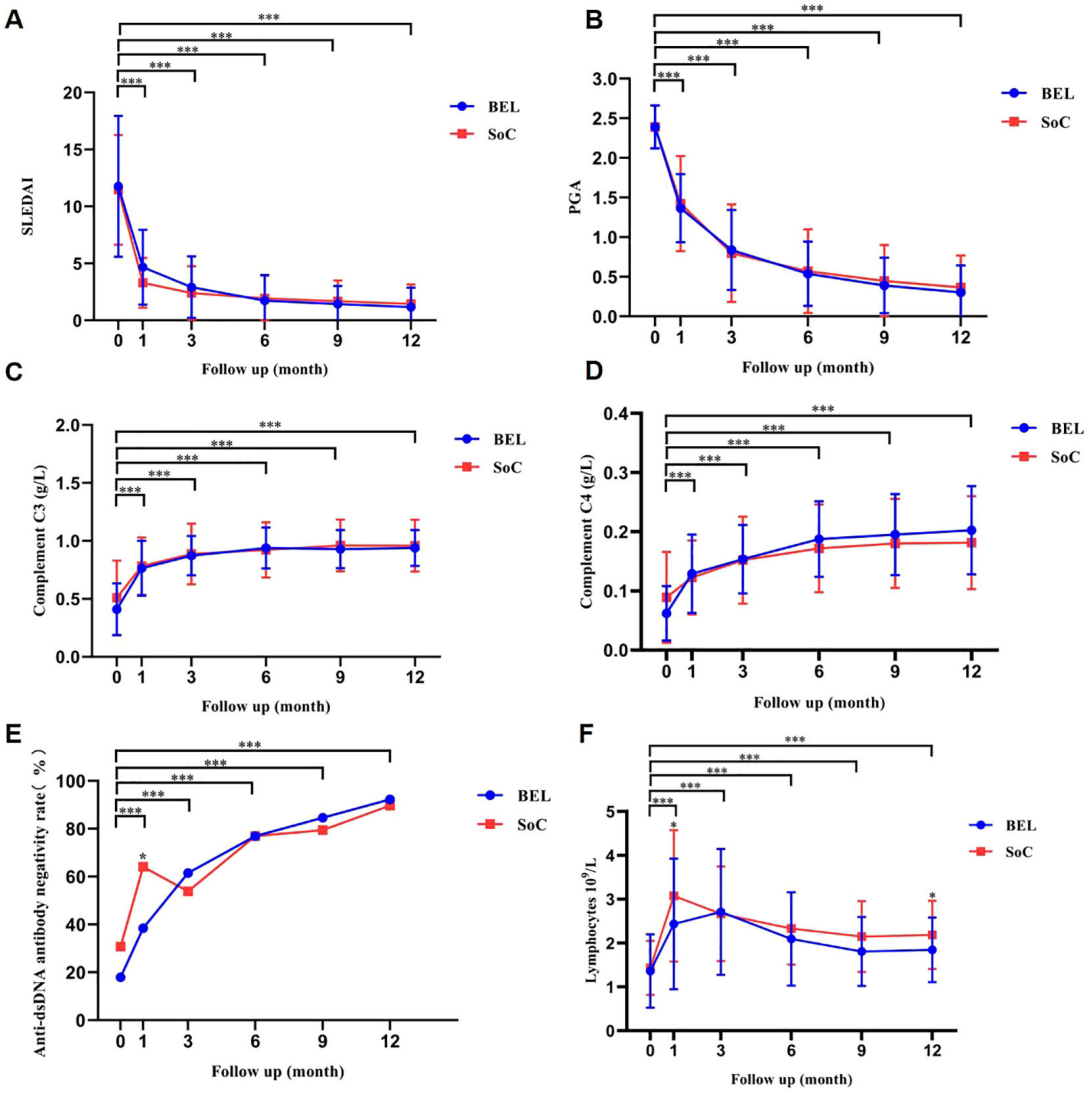
Longitudinal changes in disease activity scores, complement levels, anti-dsDNA antibody negativity, and lymphocyte counts over 12 months in belimumab and control groups. ***P<0.001 At 1, 3, 6, 9 and 12 months of treatment, there were no significant differences in SLEDAI score **(A)**, PGA score **(B)** complement C3 **(C)**, complement C4 **(D)**, anti-dsDNA antibody negativity rate **(E)**, and lymphocyte absolute value **(F)** between belimumab and control groups, except for a significant difference in anti-dsDNA antibody negativity rate at 1 month of treatment and lymphocyte at 1 and 12 months of treatment. dsDNA, double‐stranded DNA; PGA, physician global assessment; SLEDAI, Systemic Lupus Erythematosus Disease Activity Index.

Throughout the 1, 3, 6, 9, and 12-month follow-up period, patients in both groups exhibited significant improvements in SLEDAI score, PGA score, complement C3, complement C4, anti-dsDNA antibody negativity rate, and lymphocyte absolute value compared to baseline. However, no statistically significant differences were observed between the two groups except for difference in anti-dsDNA antibody negativity rate levels at 1 month of treatment and lymphocyte at 1 and 12 months of treatment (**Figures 3A–F**; **Supplementary Table 2**).

**Figure 4:**
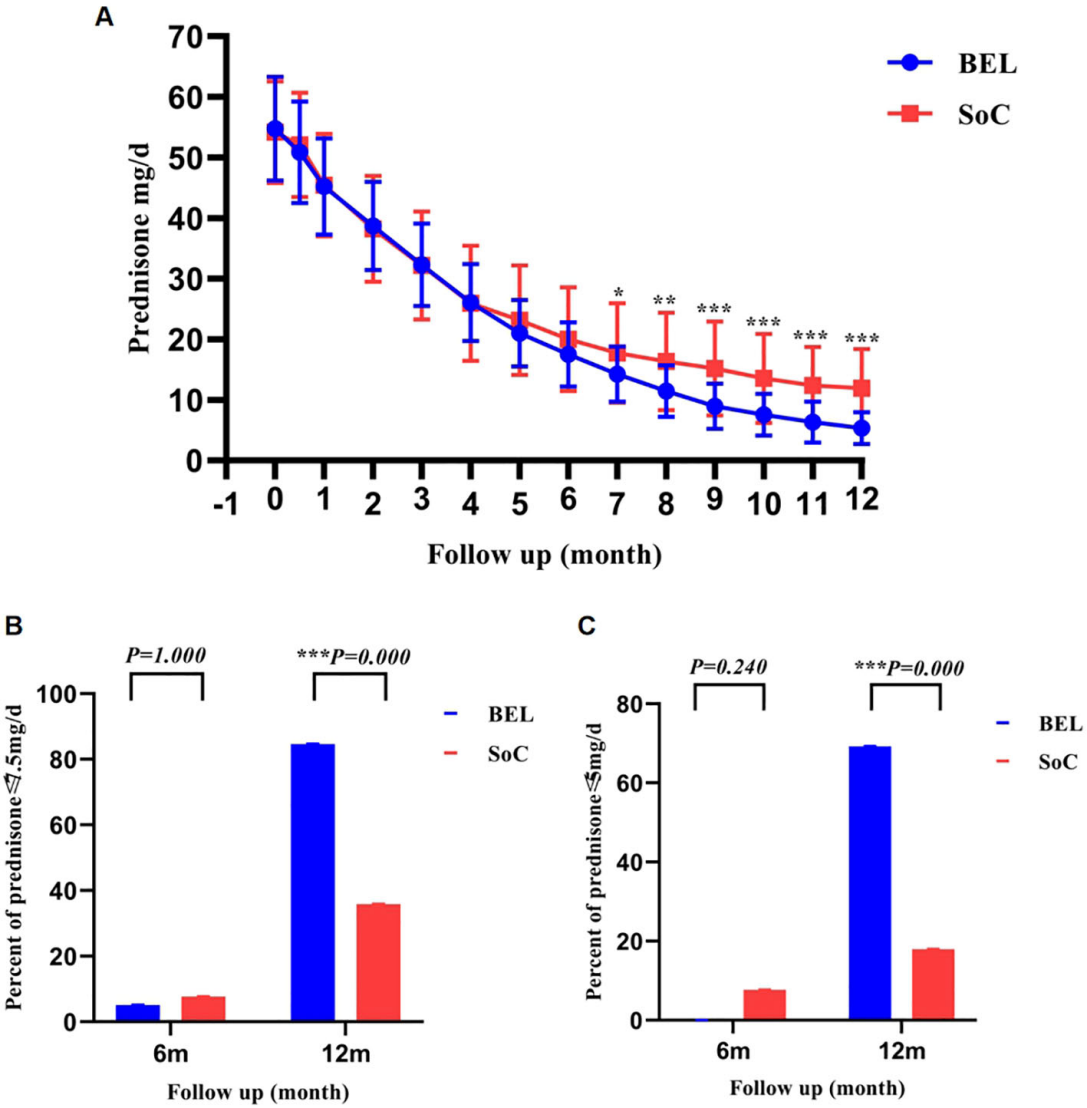
Comparison of daily prednisone doses between belimumab and control groups over 12 months. *p < 0.05; **p < 0.01;***P<0.001; **(A)** Monthly prednisone dose changes from baseline to 12 months of treatment reveal a divergence between the two groups starting at 7 months and persisting through 12 months. This difference widened with prolonged treatment duration. **(B, C)** At 6 months, there was no statistically significant difference in the proportion of patients receiving prednisone at doses of ≤7.5 mg/d and ≤5 mg/d between the two groups. However, at 12 months, the belimumab group exhibited a significantly higher proportion of patients in these dosage categories compared to the control group.

There was no statistically significant difference in daily prednisone dose between the two groups from baseline to 7 months post-treatment. However, from 7 to 12 months, the belimumab group exhibited a significantly lower daily prednisone dose compared to the control group (**Figure 4A**). Moreover, while there was no statistically significant variance in the number of prednisone doses ≤ 7.5 mg/d and ≤5mg/d at 6 months, the proportion of prednisone doses ≤ 7.5 mg/d and ≤5mg/d was notably higher in the belimumab group than in the control group at 12 months, with a statistically significant difference (**Figures 4B, C**; **Supplementary Table 3**). Our data show that both groups achieved similar control of disease activity, but adding belimumab produced a faster and more sustained reduction in daily prednisone dose. Because children vary substantially in weight, we performed a supplementary analysis using weight-adjusted prednisone thresholds to test the robustness of the steroid-sparing effect. Baseline doses in mg/kg/day were comparable between groups (BEL: 1.52 ± 0.39 vs. SoC: 1.54 [1.18, 1.94]; p=0.830). At month 12, applying derived pediatric thresholds approximating the adult LLDAS and DORIS criteria (≤0.15 mg/kg/day and ≤0.10 mg/kg/day, respectively), significantly more patients in the belimumab group met these weight-adjusted targets (69.2% vs. 33.3%, p=0.002; and 51.3% vs. 10.3%, p<0.001). As a result, a larger proportion of patients receiving belimumab attained LLDAS and DORIS despite comparable disease activity scores to the control group. These findings indicate that the principal benefit of early belimumab add-on therapy is to facilitate glucocorticoid tapering while preserving disease control.

In both the belimumab and control cohorts, children exhibited increased height, weight, and BMI from baseline to 12 months, with statistically significant differences observed. However, no statistically significant differences in height, weight, and BMI were found between the two groups at baseline and after 12 months of treatment. While the height disparity between children undergoing glucocorticoid treatment for 12 months and the median height of healthy children of similar age and sex did not reach statistical significance, significant differences were noted in weight gain and BMI compared to the normal median. Additionally, a slower annualized height growth was observed compared to the normal median.

**Figure 5:**
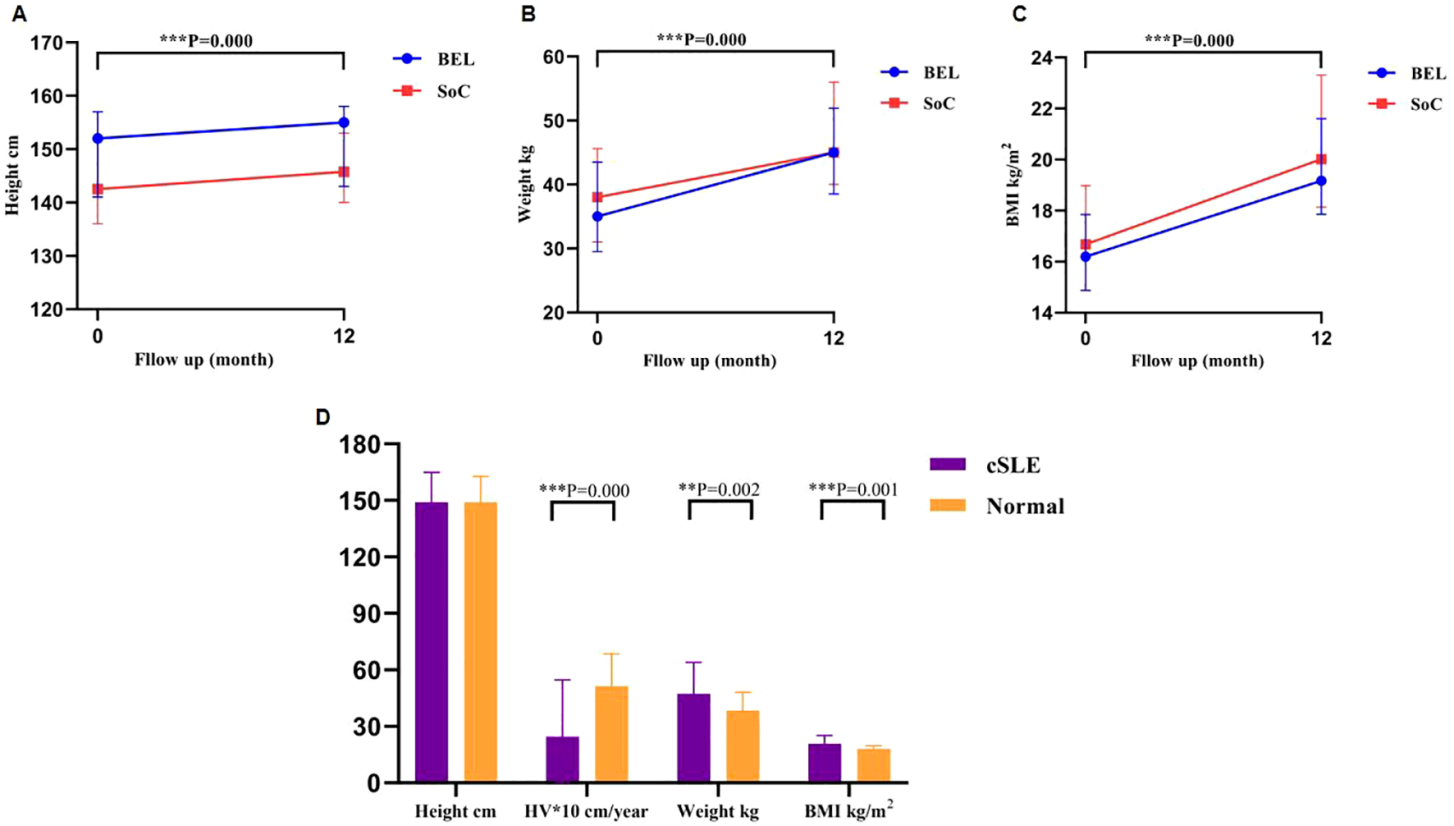
Changes in height, weight, and BMI in the belimumab and control groups at baseline and 12 months. **p < 0.01; ***P<0.001; **(A-C)** After 12 months of treatment, both groups showed increases in height, weight, and BMI compared to baseline, with no significant difference observed between the two groups. **(D)** In comparison to healthy children of corresponding age and gender, children with cSLE undergoing 12 months of prednisone treatment exhibited a statistically significant difference in weight, BMI, and HV, while height showed no statistically significant differences. BMI, body mass index.

Both the belimumab group and the control group showed statistically significant increases in children’s height, weight, and BMI from baseline to 12 months. However, there were no statistically significant differences in height, weight, and BMI between the two groups at baseline and after 12 months of treatment (**Figures 5A–C**). Children treated with glucocorticoids for 12 months did not exhibit a statistically significant difference in height compared to healthy counterparts of the same age and sex. However, they did show statistically significant increases in weight and BMI compared to the median values of healthy children, as well as a slower annualized height velocity (HV) in comparison to the healthy median (**Figures 5D**; **Supplementary Tables 4**, **5**).

**Figure 6:**
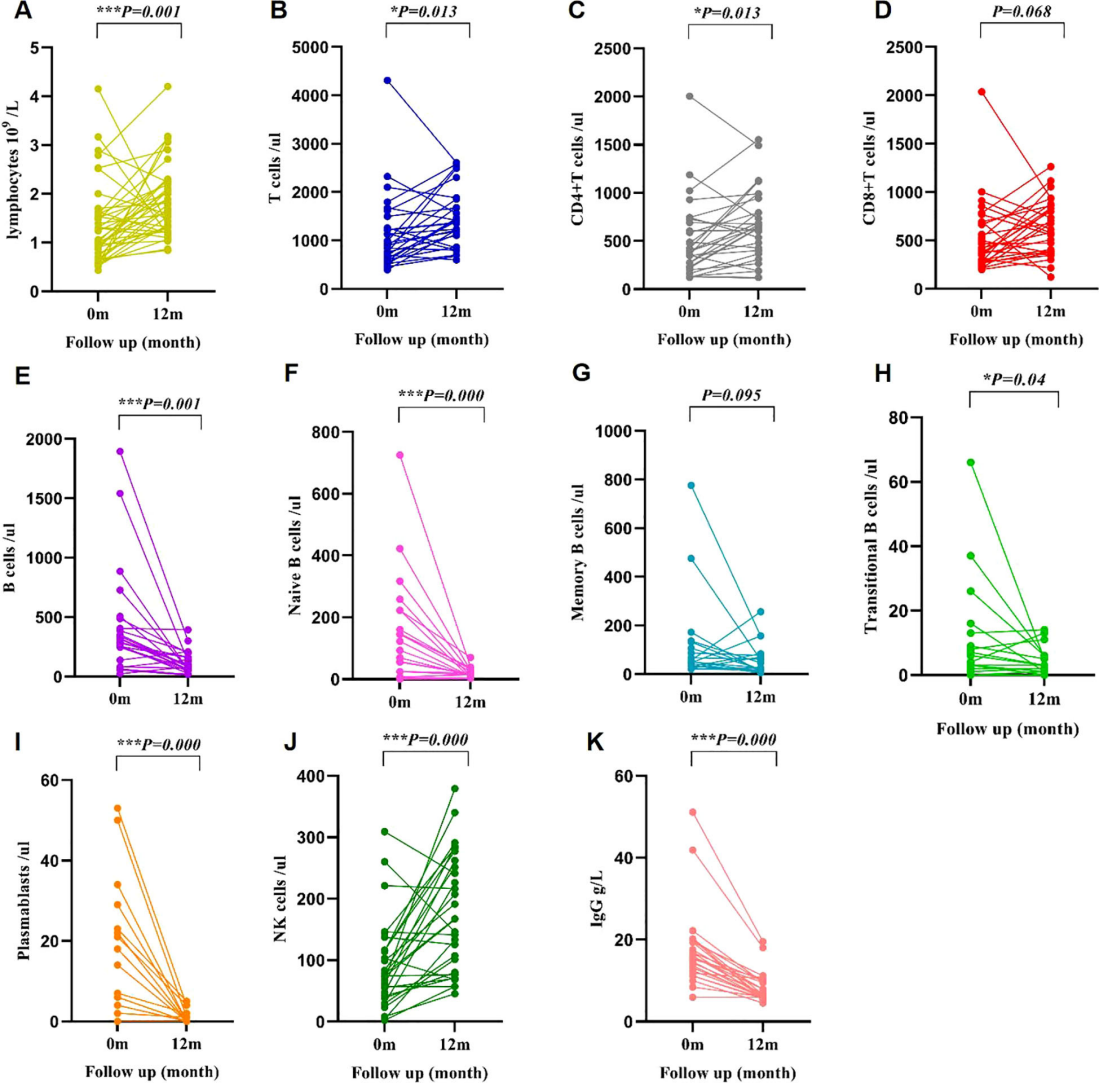
Changes in lymphocyte subsets and IgG levels in the belimumab group from baseline to 12 months. *p < 0.05; ***P<0.001; At 12 months post-treatment, there was a significant increase in lymphocytes **(A)**, T cells **(B)**, CD4+ T cells **(C)**, and NK cells **(J)** showed elevated levels compared to baseline. Conversely, B cells **(E)**, naive B cells **(F)**, transitional B cells **(H)**, plasmablasts **(I)**, and IgG **(K)** exhibited decreased levels compared to baseline. No statistically significant variance was observed in the levels of memory B cells **(G)** and CD8+ T cells **(D)** at baseline and 12 months of treatment.

To assess the impact of belimumab on immune function, immunocyte and immunoglobulin data were gathered at baseline and post 12-month treatment. The findings revealed a significant increase in lymphocytes, T cells, CD4+ T cells, and NK cells, while B cells, naive B cells, transitional B cells, plasmablasts, and IgG (exclude the effects of rituximab and intravenous immunoglobulin) exhibited a decrease after 12 months of treatment. Notably, there were no statistically significant changes observed in memory B cells and CD8+ T cells pre- and post-treatment (**Table 2**, **Figure 6**). The distribution of IgG levels at baseline and 12 months is detailed in **Supplementary Figure 1**.

**Table 2:** Changes in lymphocyte subsets and IgG levels in the belimumab group at baseline and after 12 months of treatment.

Variable	0m	12m	*P*
Lymphocytes ×10^9^/L	1.06 (0.71,1.59)	1.69 (1.30,2.24)	0.001
B cells/ul	304.00 (125.00,426.50)	82.00 (52.25,161.00)	0.000
Naive B cells/ul	122.00 (25.00,223.00)	16.00 (8.00,27.00)	0.000
Memory B cells/ul	51.00 (33.00,132.00)	25.00 (11.00,77.00)	0.095
Plasmablasts/ul	14.00 (4.00,23.00)	0.00 (0.00,1.00)	0.000
Transitional B cells/ul	4.00 (2.00,13.00)	3.00 (1.00,6.00)	0.04
IgG g/L	15.70 (12.25,19.53)	6.52 (5.89,9.77)	0.000
T cells/ul	903.00 (617.50,1379.00)	1374.00 (985.00,1769.00)	0.013
CD4+T cells/ul	393.00 (225.00,708.50)	658.41 ± 370.14	0.013
CD8+T cells/ul	403.00 (280.50,692.50)	637.90 ± 282.18	0.068
NK cells/ul	74.00 (40.00,116.00)	174.41 ± 95.03	0.000

Based on case records, 10 children in the belimumab group and 8 in the control group experienced infections, with no statistically significant difference in infection rates (p=0.591). The infections observed in the belimumab and control groups included bacterial, viral, fungal, and mycoplasma (**Supplementary Table 6**). A detailed temporal analysis of the 10 infection events in the belimumab group is provided (**Supplementary Table 7**). Only 3 events occurred when the patient’s concurrent IgG level was <7 g/L. Achieving an IgG level <7 g/L at the 12-month timepoint was not statistically associated with the occurrence of infection during follow-up (p=0.054). Infection rates per person-year, stratified by severity, are presented in **Supplementary Table 8**. The overall infection rate was 0.19 per person-year in the belimumab group versus 0.09 in the control group, with no significant difference in the rates of severe infections. Retrospective analysis of initial belimumab therapy revealed no infusion-related adverse reactions, including infusion reactions, allergies, gastrointestinal issues, neuropsychiatric abnormalities, or limb soreness.

## Discussion

cSLE is characterized by severe multi-organ involvement, high medication dosages, prolonged treatment duration, and a propensity for relapse (22, 23). The primary objective of managing SLE in children is to promptly achieve LLDAS or remission, sustain long-term remission, and minimize corticosteroid usage (24). Multiple prior clinical studies have demonstrated that administering belimumab to SLE patients, both adult and child, can lower disease activity, prevent relapses, decrease steroid dosage, and enhance the overall long-term outlook for SLE patients (25–27). The steroid dose should promptly be reduced to 5 mg/day. Initially, belimumab was reserved for patients with mild disease activity and without severe organ damage, such as renal or neurological impairment, in both adult and pediatric populations (11, 12, 28). Subsequent studies in adults and children have shown that belimumab is effective in severe or refractory disease, including LN and lupus encephalopathy (29–32). A model-based analysis of database supports early use of belimumab to improve long-term prognosis and reduce medical costs (33). A retrospective analysis of the Pisa SLE cohort similarly indicates a trend toward earlier application in recent years (34). However, these findings remain indirect, and no clear recommendations from previous studies on when to add belimumab (27, 35). Nonetheless, Yin Zhao et al. observed that initiating belimumab treatment in 33 newly diagnosed adults with SLE resulted in the highest SRI-4 rate, significant improvements in serologic markers, and SLEDAI scores compared to relapsed and refractory groups, indicating superior effectiveness of initiating belimumab treatment (14). Limited research exists on the early use of belimumab in pediatric patients. This study retrospectively analyzed children with cSLE who received initial belimumab treatment to assess the effectiveness and safety of belimumab as first-line therapy for cSLE.

The study demonstrated significantly higher rates of achieving LLDAS and DORIS in the belimumab group compared to the control group after 12 months of treatment, consistent with the findings of Yinv Gong et al. (LLDAS: Bel: Soc 75.0% vs 18.6%; DORIS Bel: Soc 34.4% vs 11.9%). However, the LLDAS and DORIS rates at 12 months in both the belimumab and control groups in the aforementioned studies in children were lower than those in our study, likely due to the presence of LN in their all cases, posing greater treatment challenges (36). A sensitivity analysis adjusting for residual imbalances, including renal/CNS involvement and background therapies, confirmed the independent association between early belimumab and superior treat-to-target outcomes. There were no differences in SLEDAI score, PGA score, or stable medication doses (antimalarials, immunosuppressants, and biologics) between the two groups at 12 months. This observation is not contradictory but rather highlights the composite nature of treat-to-target endpoints. LLDAS and DORIS are designed to reflect both effective disease control and safe management, with glucocorticoid dose as a critical component. The variation in LLDAS and DORIS rates between the groups was primarily driven by the daily prednisone dose. At the observation endpoint, children who received initial treatment plus belimumab achieved comparable disease activity scores to those on conventional therapy but experienced a faster and more pronounced reduction in glucocorticoid exposure. Chaofan Lu and colleagues’ research on the primary treatment of SLE in adult patients with belimumab revealed a higher rate of achieving LLDAS in the belimumab cohort compared to the conventional treatment group after 6 months of therapy (56.3% vs. 19.4%). However, there was no significant disparity in the rate of DORIS attainment between the two groups. This suggests that the early administration of belimumab in adults may expedite the achievement of LLDAS compared to children, potentially due to the milder nature of SLE in adults and the faster tapering of corticosteroids (15). Patients in the belimumab group achieved LLDAS and DORIS significantly faster than those in the conventional treatment group, indicating that initiating treatment with belimumab may expedite the attainment of LLDAS and DORIS.

The LLDAS and DORIS criteria use absolute prednisone dose thresholds derived from adult studies. Their application in children, with wide variations in age and body weight, presents a challenge for individualized risk-benefit assessment. We applied these standardized criteria to enable comparisons of lupus treat-to-target outcomes across age groups. This approach confirms that early belimumab increases the proportion of children reaching low-steroid treatment states. Future work to define pediatric-specific targets should consider incorporating weight-adjusted doses (mg/kg/day) to better guide personalized glucocorticoid tapering.

In this investigation, disease activity scores and laboratory tests were assessed in the belimumab and control groups at 1, 3, 6, 9, and 12 months post-treatment initiation. Children receiving belimumab exhibited enhancements in SLEDAI score, PGA score, complement C3, complement C4, anti-dsDNA antibody negativity rate, and lymphocyte across all time points compared to baseline, with statistically significant differences, consistent with findings in pediatric patients by Li Wang et al. (32). Nevertheless, no statistically significant disparities were observed between the belimumab and control groups, aligning with prior research in pediatric and adult SLE cohorts (15, 36, 37). This outcome might be attributed to the high daily prednisone dosage and gradual tapering in the conventional treatment group.

Long-term glucocorticoid use poses increased side effects, particularly in pediatric patients (38, 39). During the management of SLE, particular attention should be given to drug-related adverse effects. It is advisable to taper and discontinue glucocorticoids promptly once the disease is under control. Previous research on cSLE has demonstrated that belimumab can contribute to lowering prednisone dosages in children with active and refractory cSLE (31, 32). Analysis of prednisone dosages in the two groups indicated no statistically significant difference in daily prednisone dose during the period of induced remission. However, after 7 months of treatment, the belimumab group exhibited a lower daily prednisone dose compared to the conventional treatment group. Hashimoto et al. demonstrated that glucocorticoid tapering was notably accelerated in the cSLE group receiving early belimumab treatment at 6 months compared to the control group, aligning with our study’s results (40). Despite the belimumab group’s lower and rapidly tapered prednisone dose, no statistical disparity was observed between the two groups in terms of clinical symptom improvement and immunological parameters within the same timeframe. Over time, the discrepancy in daily prednisone dose between the two groups of pediatric patients became more pronounced, suggesting a swifter reduction rate in the belimumab group. Chiara Tani and colleagues investigated a cohort of adults with SLE from Pisa who did not have LN. Patients were stratified into two cohorts based on prior exposure to immunosuppressive therapy before initiating belimumab. The study revealed that patients who had not undergone immunosuppressive therapy required significantly lower daily doses of prednisone at 6 and 12 months, indicating a potential benefit of early initiation of belimumab in reducing glucocorticoid dosage (34). Although the proportion of patients with prednisone doses ≤7.5mg/day and ≤5mg/day did not differ between the two groups after 6 months of treatment, it was notably higher in the belimumab group compared to the conventional treatment group after 12 months of treatment, consistent with findings in children from Yinv Gong’s study (12-month prednisone dosage ≤7.5mg/day: 82.9% vs. 30.4%; ≤5mg/day: 42.9% vs. 19.6%) (36). In the context of cSLE, the primary advantage of initiating treatment with belimumab lies in sustaining remission, facilitating a quicker reduction in glucocorticoid dosage with stabilization, and mitigating glucocorticoid-related complications.

At 12 months, the belimumab group exhibited a lower daily prednisone dose compared to the control group. However, there were no statistically significant differences in height, weight, and BMI between the two groups. This lack of significance may be attributed to minimal prednisone variations and the brief duration of observation. Children with cSLE undergoing 12 months of steroid therapy displayed significantly elevated body weight and BMI, along with markedly reduced HV compared to healthy peers of similar age and gender. These findings underscore the detrimental impact of glucocorticoid administration on children’s growth and development, potentially leading to obesity and short stature. Nevertheless, glucocorticoid are presently the primary treatment approach for SLE. The primary objectives of lupus therapy are to manage the disease promptly and reduce glucocorticoid dosages. The utilization of belimumab aids in achieving these objectives. Over time, it is hypothesized that the low-steroid-dose belimumab group will exhibit therapeutic benefits in terms of height, weight, and BMI. Although the difference in height at 12 months of treatment compared to normal children was not statistically significant, the reduced HV in children receiving prednisone therapy may lead to a gradual lag in height if high-dose prednisone is continued.

Belimumab inhibits B cell proliferation and differentiation, induces B cell apoptosis, and suppresses autoantibody production by binding to B lymphocyte stimulating factor (BLyS) (41). A retrospective analysis in this study demonstrated that children treated with belimumab for 12 months exhibited increased lymphocytes, T cells, and NK cells, along with decreased B cells compared to baseline. Specifically, naive B cells, plasmablasts, and transitional B cells decreased, accompanied by a reduction in IgG levels, consistent with existing literature on belimumab therapy with children and adult (11, 31, 42). The decrease in B cells and IgG levels likely reflects effective suppression of pathogenic autoantibody production and aligns with clinical treatment response. Importantly, this immunologic change did not lead to an increased risk of infection. Pediatric patients on belimumab should be monitored for infections. Immunoglobulin supplementation may be indicated based on infection severity and IgG levels.

Upon retrospective analysis, no adverse reactions, such as infusion reactions, allergic reactions, gastrointestinal and neuropsychiatric abnormalities, limb soreness, or neutropenia, were observed in children treated with belimumab at our center. This indicates that the drug can be safely administered in the early stages of cSLE. While infections may arise in children receiving initial belimumab therapy, they are generally not severe or uncontrollable. Importantly, none of the patients discontinued belimumab due to infection.

Our study has several limitations that warrant consideration. As a retrospective observational study, our findings are subject to potential confounding by indication. The clinical decision to initiate belimumab early may have been influenced by disease severity, organ involvement, or physician’s anticipation. To reduce imbalance in measured confounders, we applied PSM using key baseline variables (age, gender, SLEDAI, PGA, initial steroid dose), and most baseline characteristics were well-balanced (SMD<0.1) (**Table 1**). Nonetheless, unmeasured or residual confounding—such as nuanced physician judgment, socioeconomic factors, or specific serological profiles—may persist. Despite this inherent limitation of observational research, the significantly improved outcomes in the belimumab group, including faster achievement of treat-to-target endpoints, accelerated steroid tapering, and favorable immunomodulatory effects, are persuasive and indicate a potential benefit of early biologic intervention. These real−world data therefore provide a strong rationale for prospective, randomized controlled trials designed to compare early versus delayed belimumab initiation in cSLE. Our statistical approach used pointwise comparisons and therefore did not model individual trajectories over time. Future studies should apply longitudinal modeling in larger cohorts to delineate treatment-response trajectories more precisely. As a single-center retrospective study, our findings may be subject to selection bias and may not be fully generalizable to other settings or populations. Although the sample size was adequate for an initial exploratory analysis, it remains modest and may limit statistical power to detect differences in secondary outcomes or rare adverse events. the follow-up duration, while adequate for assessing short- to medium-term outcomes, does not allow evaluation of long-term efficacy, safety, or cumulative organ damage. The application of adult-derived treat-to-target criteria (LLDAS/DORIS) with fixed glucocorticoid thresholds may not fully reflect pediatric treatment goals, especially in younger children with variable body weights. While longitudinal weight-adjusted dosing analysis was limited by unavailable serial weight data, a sensitivity analysis at 12 months using derived pediatric thresholds (≤0.15 mg/kg/day for LLDAS-equivalent and ≤0.10 mg/kg/day for DORIS-equivalent) confirmed significantly higher attainment in the belimumab group (both p<0.01). This reinforces that the observed steroid-sparing effect is substantial even when adjusted for body weight.

In summary, incorporating belimumab with the conventional treatment of glucocorticoids, immunosuppressants, and HCQ at the onset of cSLE can expedite the attainment of LLDAS and remission in pediatric patients, facilitating glucocorticoids tapering. Despite its favorable safety profile and limited adverse effects, early adjunctive use of belimumab holds promise for enhancing outcomes in cSLE. Nonetheless, caution is warranted due to its potential impact on immune function, manifested by reduced B cells and IgG levels, necessitating close monitoring for infection susceptibility. This investigation introduces a novel therapeutic approach and lays the groundwork for standardized cSLE management. Future research should focus on enlarging the sample, conducting detailed analyses of belimumab effectiveness in cSLE across various regimens and timings, and refining the therapeutic strategy for cSLE.

## Data Availability

The original contributions presented in the study are included in the article/**Supplementary Material**. further inquiries can be directed to the corresponding author.
