# Supplementary material for: Effectiveness and safety analysis of initial treatment with belimumab in childhood-onset systemic lupus erythematosus
Source: Front Immunol. 2026 Mar 16;17:1769627. doi: 10.3389/fimmu.2026.1769627 (PMC13033727; doi:10.3389/fimmu.2026.1769627)
Supplement: Supplementary file 1 [file DataSheet1.docx]

Table 1 Decomposition of LLDAS and DORIS components at 12 months in the belimumab and control groups

|  | BEL | | SoC | |
| --- | --- | --- | --- | --- |
|  | N | % | N | % |
| LLDAS | 31/39 | 79.49 | 14/39 | 35.9 |
| SLEDAI≤4 | 37/39 | 94.87 | 38/39 | 97.44 |
| PGA≤1 | 39/39 | 100 | 39/39 | 100 |
| Prednisone dose ≤7.5 mg/day | 33/39 | 84.62 | 14/39 | 35.9 |
| DORIS | 18/39 | 46.15 | 5/39 | 12.82 |
| Clinical SLEDAI=0  (irrespective of serological parameters) | 25/39 | 64.1 | 20/39 | 51.28 |
| PGA<0.5 | 24/39 | 61.54 | 23/39 | 58.97 |
| Prednisone dose ≤ 5 mg/day | 27/39 | 69.23 | 7/39 | 17.95 |
| No disease activity in major organs | 39/39 | 100 | 39/39 | 100 |
| No new disease activity | 39/39 | 100 | 39/39 | 100 |
| Standard maintenance doses of immunosuppressants and biologics | 39/39 | 100 | 39/39 | 100 |

Abbreviations: BEL, belimumab; DORIS, Definitions of Remission in Systemic Lupus Erythematosus; PGA, physician global assessment; LLDAS, lupus low disease activity status; SLEDAI, Systemic Lupus Erythematosus Disease Activity Index; SoC, standard of care.

Table 2 Disease activity scores and indicators in belimumab group and control group during follow-up

|  |  | SLEDAI | PGA | Complement C3 g/L | Complement C4 g/L | Anti-dsDNA antibody negativity rate % | Lymphocyte 10^9^/L |
| --- | --- | --- | --- | --- | --- | --- | --- |
| 0m | BEL | 11.77±6.19 | 2.5(2.2,2.5) | 0.37(0.23,0.63) | 0.043(0.03,0.085) | 17.95 | 1.06(0.71,1.59) |
|  | SoC | 11.46±4.82 | 2.5(2.2,2.6) | 0.39(0.28,0.64) | 0.054(0.039,0.14) | 30.77 | 1.43±0.62 |
|  | *P* | 0.807 | 0.827 | 0.252 | 0.149 | 0.187 | 0.261 |
| 1m | BEL | 4(2,8) | 1.5(1.2,1.5) | 0.76±0.24 | 0.12(0.086,0.171) | 38.46 | 2(1.52,3.02) |
|  | SoC | 4(2,4) | 1.6(1.0,1.8) | 0.79(0.6,0.91) | 0.121(0.076,0.154) | 64.10 | 2.83(1.9,3.7) |
|  | *P* | 0.107 | 0.121 | 0.704 | 0.664 | 0.023 | 0.02 |
| 3m | BEL | 2(2,4) | 1(0.5,1.2) | 0.87±0.17 | 0.142(0.113,0.17) | 61.54 | 2.47(1.77,3.11) |
|  | SoC | 2(0,4) | 0.8(0.0,1.2) | 0.86(0.72,0.99) | 0.146(0.101,0.192) | 53.85 | 2.67±1.08 |
|  | *P* | 0.361 | 0.734 | 0.948 | 0.818 | 0.492 | 0.742 |
| 6m | BEL | 2(0,2) | 0.5(0.2,0.8) | 0.89(0.82,1.03) | 0.178(0.147,0.223) | 76.92 | 1.9(1.32,2.72) |
|  | SoC | 2(0,2) | 0.5(0.0,1.0) | 0.92±0.24 | 0.159(0.138,0.2) | 76.92 | 2.33±0.83 |
|  | *P* | 0.407 | 0.947 | 0.576 | 0.168 | 1 | 0.113 |
| 9m | BEL | 2(0,2) | 0.5(0.0,0.8) | 0.93±0.17 | 0.20±0.07 | 84.62 | 1.81±0.79 |
|  | SoC | 2(0,2) | 0.3(0.0,1.0) | 0.91(0.8,1.09) | 0.17(0.13,0.218) | 79.49 | 2.15±0.81 |
|  | *P* | 0.594 | 0.585 | 0.757 | 0.259 | 0.555 | 0.062 |
| 12m | BEL | 0(0,2) | 0.2(0.0,0.7) | 0.9(0.83,1.04) | 0.186(0.15,0.259) | 92.31 | 1.69(1.3,2.24) |
|  | SoC | 1(0,2) | 0.2(0.0,0.8) | 0.95(0.81,1.03) | 0.157(0.123,0.215) | 89.74 | 2.19±0.78 |
|  | *P* | 0.371 | 0.444 | 0.857 | 0.114 | 1 | 0.034 |

Table 3 Changes in prednisone dose during follow-up and the proportion of prednisone ≤7.5 mg/d and ≤5 mg/d at 6 and 12 months of treatment

|  | Daily prednisone dose mg/L | | | Proportion of prednisone≤7.5mg/L % | | | Proportion of prednisone≤5mg/L % | | |
| --- | --- | --- | --- | --- | --- | --- | --- | --- | --- |
|  | BEL | SoC | *P* | BEL | SoC | *P* | BEL | SoC | P |
| 0m | 60(50,60) | 60(50,60) | 0.668 |  |  |  |  |  |  |
| 0.5m | 55(45,55) | 55(50,60) | 0.374 |  |  |  |  |  |  |
| 1m | 50(40,50) | 50(40,50) | 0.790 |  |  |  |  |  |  |
| 2m | 40(35,45) | 40(30,45) | 0.886 |  |  |  |  |  |  |
| 3m | 35(27.5,40) | 32.18±8.91 | 0.972 |  |  |  |  |  |  |
| 4m | 25(20,30) | 25.96±9.49 | 0.864 |  |  |  |  |  |  |
| 5m | 21.01±5.52 | 23.14±9.06 | 0.215 |  |  |  |  |  |  |
| 6m | 17.5(15,20) | 20.00±8.55 | 0.155 | 5.13 | 7.69 | 1.000 | 0.00 | 7.69 | 0.240 |
| 7m | 15(12.5,17.5) | 17.72±8.24 | 0.035 |  |  |  |  |  |  |
| 8m | 12.5(7.5,15) | 16.34±8.05 | 0.002 |  |  |  |  |  |  |
| 9m | 8.96±3.71 | 15(10,17.5) | 0.000 |  |  |  |  |  |  |
| 10m | 7.5(5,10) | 13.53±7.37 | 0.000 |  |  |  |  |  |  |
| 11m | 5(3.75,7.5) | 12.37±6.37 | 0.000 |  |  |  |  |  |  |
| 12m | 5(2.5,7.5) | 11.92±6.48 | 0.000 | 84.62 | 35.90 | 0.000 | 69.23 | 17.95 | 0.000 |

Table 4 Height, weight, and BMI of children in the belimumab and control groups at 6 and 12 months of treatment

|  |  | Height | Weigt | BMI |
| --- | --- | --- | --- | --- |
| 0m | BEL | 149.04±14.73 | 35（28.5.48） | 16.19(14.55,19.14) |
|  | SoC | 142.86±19.57 | 40.74±16.83 | 16.68(14.67,20.42) |
|  | *P* | 0.155 | 0.808 | 0.712 |
| 12m | BEL | 150.59±14.06 | 47.28±15.27 | 19.17(17.60,22.96) |
|  | SoC | 146.14±18.30 | 50.45±19.54 | 20.02(17.73,24.21) |
|  | *P* | 0.277 | 0.44 | 0.634 |

Abbreviations: BMI, body mass index.

Table 5 Comparison of height, weight and BMI of cSLE children treated with prednisone for 12 months with healthy children of the same age and sex

|  | cSLE （used prednisone for 12m） | Median values of healthy children | *P* |
| --- | --- | --- | --- |
| Height | 149.1±15.83 | 151.72（142.32，160） | 0.836 |
| HV | 2（0.625，4） | 5.9（3.4，6.2） | 0.000 |
| Weight | 45（36，56） | 38.54±9.47 | 0.002 |
| BMI | 19.48（17.64，23.31） | 18.06（16.97，19.62） | 0.001 |

Abbreviations: cSLE, childhood-onset systemic lupus erythematosus; HV, height velocity.

Table 6 The types of infections observed in the belimumab and control groups

| Types of infections | BEL | SoC |
| --- | --- | --- |
| Bacteria | 4 | 2 |
| Virus | 4 | 3 |
| Fungi | 0 | 2 |
| Mycoplasma | 2 | 1 |

Table 7 Detailed analysis of infection events in the belimumab group

|  | Type of infection | Time of infection after treatment (m) | IgG g/L |
| --- | --- | --- | --- |
| P1 | Bacteria | 30 | 7.05 |
| P2 | Mycoplasma | 7 | 9.7 |
| P3 | Bacteria | 10 | 9.97 |
| P4 | Virus | 23 | 14.6 |
| P5 | Virus | 21 | 16.6 |
| P6 | Virus | 17 | 19.5 |
| P7 | Bacteria | 11 | 7.14 |
| P8 | Mycoplasma | 19 | 4.43 |
| P9 | Virus | 11 | 5.19 |
| P10 | Bacteria | 12 | 6.2 |

Table 8 Infection rates per person-year in the belimumab and control groups

|  | BEL | SoC | P |
| --- | --- | --- | --- |
| Total person-years of follow-up | 51.67 | 89.67 |  |
| Total infection events | 10 | 8 |  |
| Infection rates  (per person-year) | 0.19 | 0.09 | 0.074 |
| Non-severe infections  (per person-year) | 0.12 | 0.06 | 0.168 |
| Severe infections  (per person-year) | 0.08 | 0.03 | 0.26 |


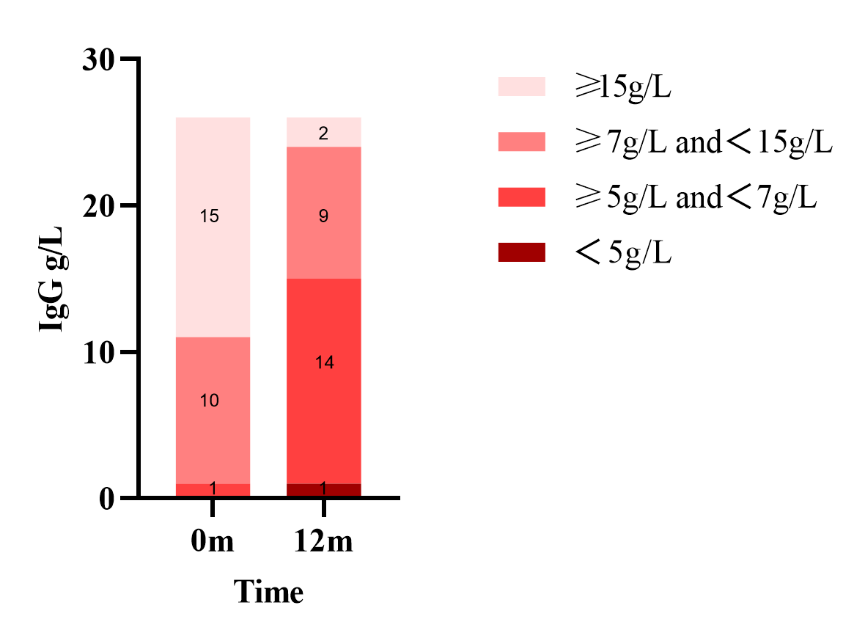


Figure 1 Distribution of IgG levels of the belimumab group at baseline and 12 months.
